# Biomechanical assessment of polyaxial combination-hole locking plates for screw push-out strength and three-point bending

**DOI:** 10.3389/fvets.2026.1806461

**Published:** 2026-07-13

**Authors:** Brittany Losey, Karl Maritato

**Affiliations:** MedVet Cincinnati, Cincinnati, OH, United States

**Keywords:** combination-hole, locking plates, polyaxial, push-out strength, three-point bending

## Abstract

**Introduction:**

Polyaxial locking plate systems and combination-hole designs expand implant adaptability during fracture fixation; however, limited data exist regarding their mechanical behavior. This study evaluated screw push-out strength and three-point bending performance of a 3.5-mm polyaxial combination-hole locking plate.

**Methods:**

Axial screw push-out testing was performed on 31 screws inserted into three 12-hole plates and tightened to 2.0 Nm. Screws were loaded at 0° angulation under quasi-static conditions until failure. Three-point bending was conducted following ASTM F382 using a 90-mm support span to determine flexural stiffness, flexural modulus, flexural strength, and maximum load to failure. Area moment of inertia (AMI) was estimated from bending data to provide geometric context for structural performance. Data were reported descriptively. No statistical comparisons were performed.

**Results:**

Mean screw push-out forces ranged from 2,839 ± 471 N to 3,119 ± 274 N. Three-point bending yielded flexural strengths of 641–697 MPa, flexural moduli of 55,655–63,732 MPa, flexural stiffness values of 133–141 N/mm, and maximum loads to failure of 0.63–0.65 kN. Estimated AMI values ranged from 32.4 to 38.0 mm^4^. Results indicate that the combination-hole configuration did not adversely affect the locking mechanism of the plate under the conditions tested. Mechanical responses were consistent across plates, demonstrating uniform screw–plate engagement and predictable bending behavior.

**Discussion:**

Observed screw retention and bending responses were uniform across plates and fell within ranges reported for other veterinary locking plate systems. Results represent baseline mechanical characteristics of the screw-plate interface and plate bending behavior under controlled testing conditions. Interpretation is limited by testing at 0° angulation, a plate-only construct, and differences in testing methodologies across previously reported studies.

**Conclusion:**

This study provides foundational mechanical data for a 3.5-mm polyaxial combination-hole locking plate, demonstrating that incorporation of a combination-hole interface does not compromise locking screw engagement or bending behavior under baseline testing conditions. Further investigation using standardized test configurations, cyclic loading, fracture-gap models, and variable screw angulation is warranted to define comparative performance and clinical relevance.

## 1 Introduction

The development of locking bone plate systems has shifted the approach of fracture management from absolute rigidity and reconstruction to a biological approach to minimize further injury to the fracture site (1, 2). The advantages of locking plates include preserving the periosteal blood supply, improved construct stability, decreased risk of disrupting fracture hematoma (when applied using minimally invasive techniques), reduced need for plate contouring, and larger core diameter of locking screws which increase bending and shear strength (1–5). Polyaxial locking plates utilize a locking mechanism that allows variable screw angles, up to 15 degrees of angulation, to improve load distribution and stability of fracture fixation (1, 6–8). Having angulation ability also allows placement of screws in locations where traditional 90-degree screws would be detrimental to the repair, such as avoiding joint surfaces, fracture lines, or previously placed implants (9).

The advancement of locking plates to include combination holes, combining conventional and threaded holes, allows for additional flexibility and adaptability during fracture fixation (10). Combination holes incorporate both threaded and non-threaded regions, enabling placement of cortical screws for interfragmentary compression when indicated. This design allows a single plate to be used in compression, hybrid, or bridging configurations within a single implant system (8, 11).

Previous veterinary orthopedic studies have investigated the biomechanical properties of polyaxial locking plates for the effects of screw angulation and torque on push-out strength and construct failure (2, 4, 6, 12). For example, Bufkin et al. (2) demonstrated that increased angulation and reduced insertion torque decrease push-out strength, while Kaczmarek et al. (7) showed that screw angulation negatively affects construct stability in a fracture-gap model. More recently, Martínez-Fortún et al. (9) showed that increasing screw angulation adversely influences the mechanical properties of a polyaxial locking plate fixation model. Altogether, these studies highlight the importance of screw-plate interface mechanics and testing configuration.

Human orthopedic literature reports similar results that mechanical behavior of variable-angle locking systems depend on plate material, geometry, and the design of the screw-plate interface. Glowacki et al. (13, 14) demonstrated thread engagement, off-axis insertion, and repeated insertions can reduce the mechanical performance of the polyaxial locking interface, highlighting the importance of locking mechanism design when interpreting load to failure and bending behavior. A review of monoaxial versus polyaxial locking plates further described the influence of implant design and testing configuration on mechanical outcomes (15).

The integration of a polyaxial locking mechanism within a combination-hole configuration was intended to combine the versatility of variable-angle locking screw placement with the flexibility of a combination-hole design capable of accepting either locking or cortical screws. This design may allow a single implant system to be used across a wider range of fracture configurations while reducing the need for multiple implant inventories. Despite the growing literature, baseline mechanical data for veterinary polyaxial combination-hole plates remain limited. Existing studies differ in test configuration, including push-out versus construct-level testing, three-point versus four-point bending, isolated plate versus fracture gap models, and variation in screw angulation. The objective of this study was to characterize the mechanical behavior of 3.5 mm combination-hole locking bone plates (InVictos Veterinary Orthopedics, USA) under controlled conditions for axial push-out screw strength and three-point bending properties. These findings are presented within the context of previously reported veterinary locking plate properties; however, differences in methodology limit direct comparison (2, 6, 12, 16).

We hypothesized that the 3.5-mm combination-hole locking plate (InVictos) would have consistent screw-plate interface holding strength and reproducible bending behavior under controlled loading conditions.

## 2 Materials and methods

**Figure 1:**
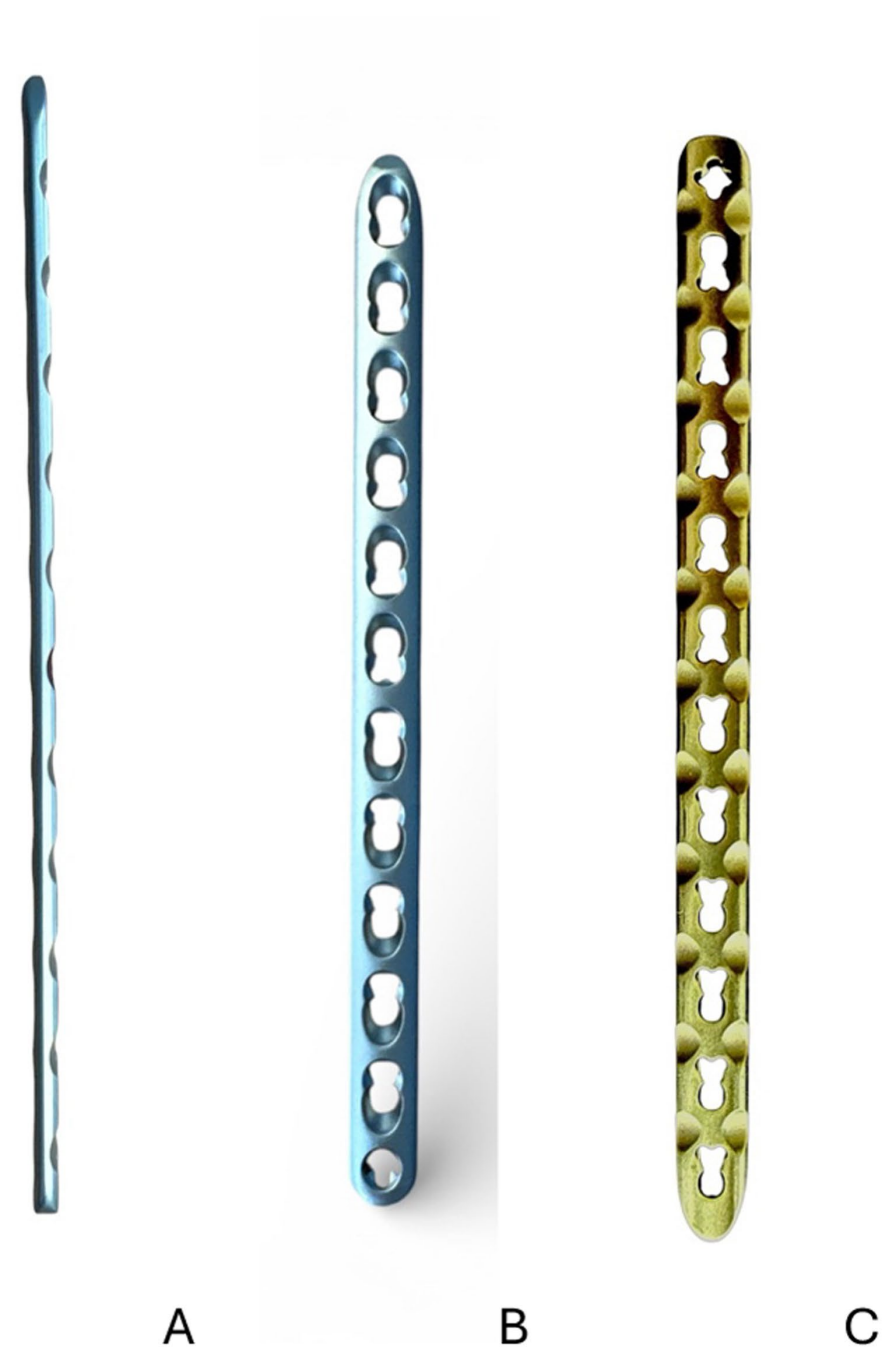
A 3.5-mm polyaxial combination-hole locking plate (InVictos). **(A)** Lateral view, **(B)** Dorsal view, **(C)** ventral view showing combination holes with locking and cortical regions. Plates were 12-hole, 316LVM stainless steel constructs.

**Figure 2:**
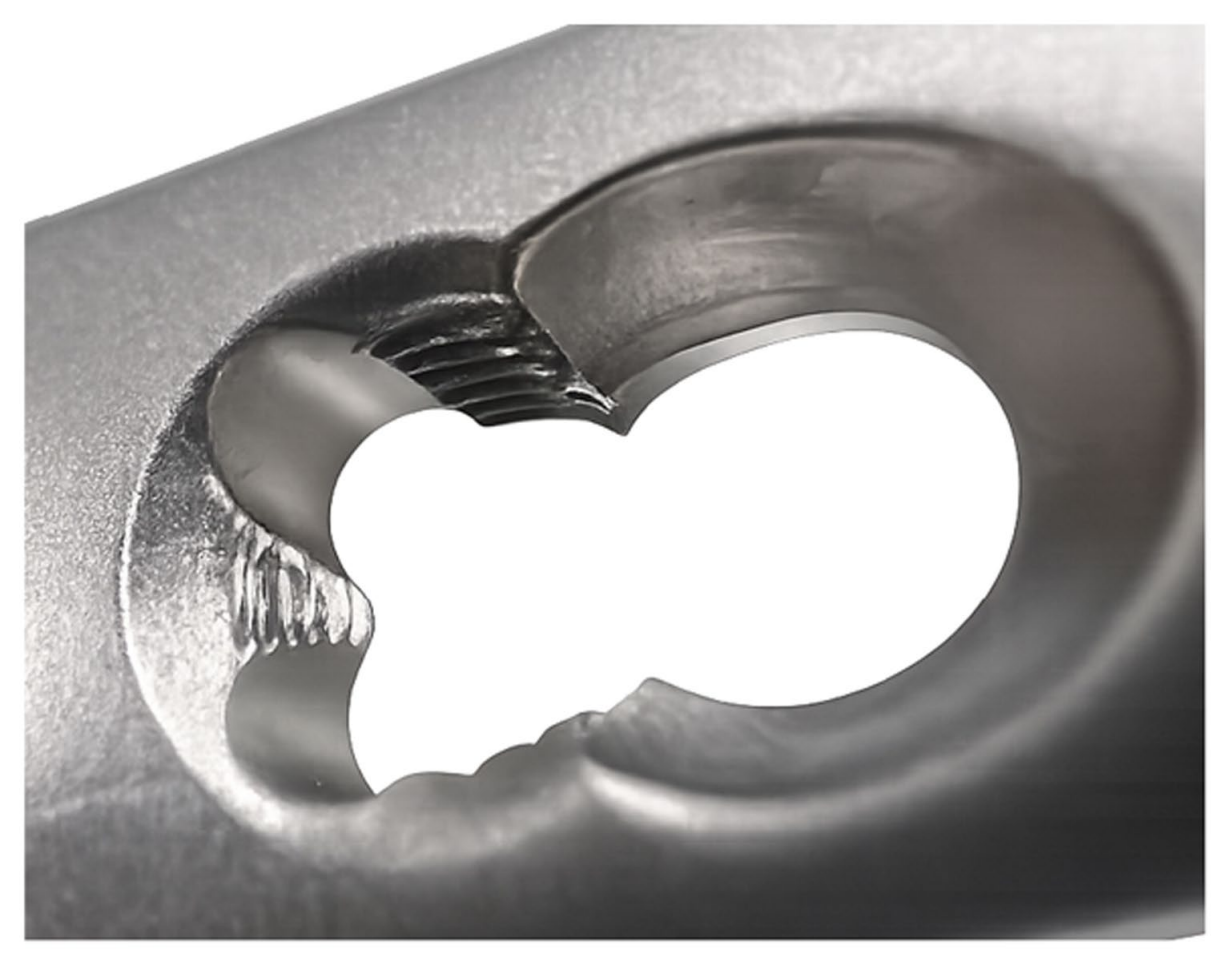
Close-up view of a combination-hole within the 3.5-mm polyaxial locking plate, demonstrating the threaded locking region and adjacent compression slot.

A 3.5-mm polyaxial combination-hole locking plate (InVictos) was evaluated in this study (Figure 1). The plate was manufactured from 316LVM stainless steel and features combination holes incorporating both threaded regions for locking screw engagement and non-threaded regions to permit placement of standard cortical screws. The locking mechanism utilized a threaded polyaxial interface in which double helix locking screws engage with corresponding threads within the plate hole (Figure 2). This differs from other systems that utilize friction-based locking, thread-cutting design, or locking caps. Compared to traditional combi-hole designs (e.g., Synthes Locking Compression Plate) (8), which incorporate fixed-angle threaded holes and dynamic compression holes, the present system integrates a variable-angle threaded interface within the combination-hole configuration. All plates tested were 12-hole constructs of identical dimensions and configuration.

### 2.1 Experiment 1: screw push-out test

**Figure 3:**
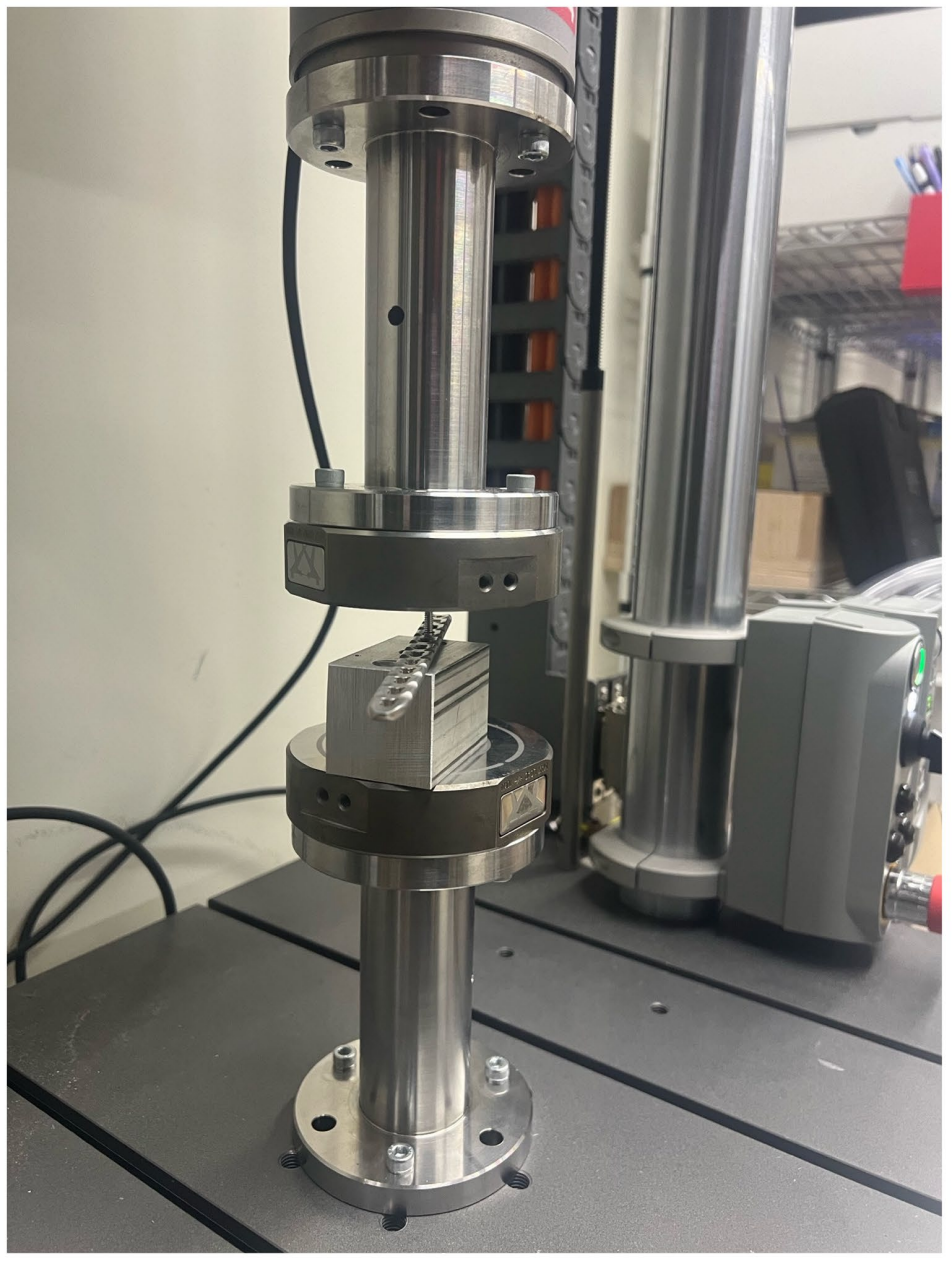
Screw push-out test setup showing axial loading of locking screws inserted at 0° angulation into locking holes until displacement.

This experiment quantified axial screw retention strength by measuring the force required to displace locking screws from individual polyaxial combination holes. No universally accepted standard exists for screw push-out testing of locking plate systems. Accordingly, the methodology was adapted from previously described veterinary orthopedic studies evaluating screw–plate interface strength and the effects of angulation and insertion torque on mechanical performance (2, 7, 12, 17). Three 12-hole plates were used, and 31 screws (10–11 per plate) were tested using an axial loading setup. The terminal hole was excluded as it does not contain a full combination-hole locking interface. The maximum axial force (N) required to dislodge each screw from its corresponding locking hole was measured. Each screw was inserted using a torque driver (Utica KT-30) calibrated at 2.0 Nm. Although AO guidelines recommend 1.5 Nm for 3.5-mm locking screws, a higher torque was selected to ensure consistent thread engagement within the polyaxial mechanism achievable in a clinical setting, as supported by findings that torque magnitude influences push-out strength (2, 17). Screws were inserted with the intention of achieving 0° (monoaxial) alignment relative to the plate. Insertion was performed manually without the use of a guiding jig; however, care was taken to maintain perpendicular alignment during screw placement. The plate was supported in a custom fixture to allow isolated axial loading of the screw. Following insertion, axial push-out testing was performed using an Instron mechanical testing machine under displacement-controlled quasi-static loading at a rate of 1 mm/min until the peak force required to dislodge the screw was observed (Figure 3). The mode of failure was observed at the screw-hole interface, thread stripping, or plate deformation. A load–displacement curve was recorded for each screw to identify subtle failure events in addition to the average screw push-out force. Statistical analysis was performed using one-way analysis of variance (ANOVA) to compare screw push-out forces among the three tested plates. Individual screw push-out measurements were treated as observations for exploratory comparison of screw–plate interface performance. Statistical significance was defined as *p* < 0.05.

### 2.2 Experiment 2: plate construct three-point bending

Three, 12-hole 3.5-mm polyaxial combination-hole plates (InVictos, Plates 1–3) were subjected to a three-point bending test to determine the flexural strength, flexural stiffness, flexural modulus, and maximum flexural stress of each plate under controlled loading conditions. The bending load is usually among the dominant forces experienced by fracture fixation plates under *in vivo* conditions. Three-point bending was selected to explore the flexural strength of the plate because it provides a conservative, reproducible, and clinically relevant assessment of bending-dominated loading in long, slender polyaxial combination-hole plates, consistent with established orthopedic implant testing standards (11, 18, 19).

**Figure 4:**
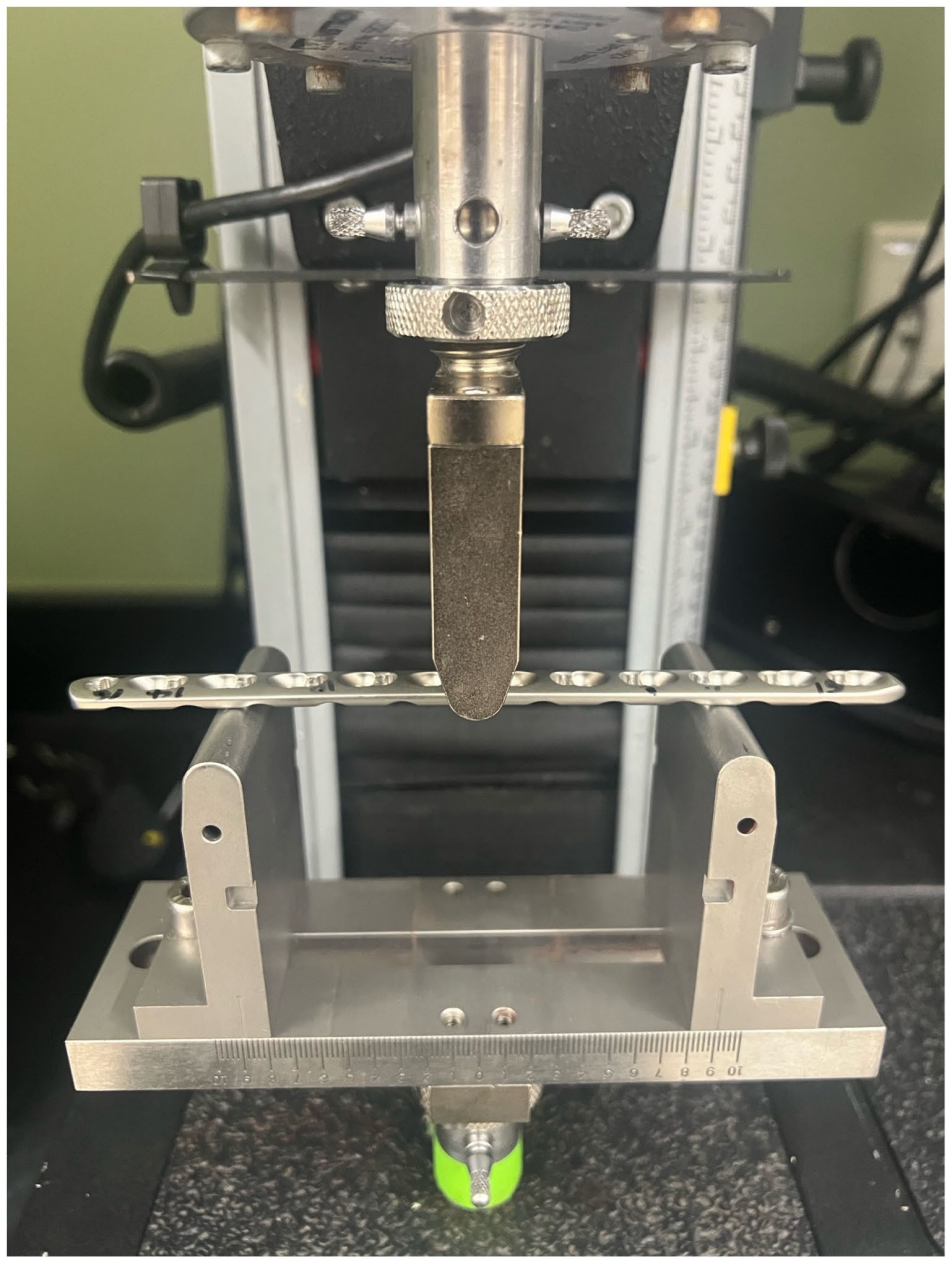
Mechanical testing configuration and load application for three-point bending.

Following the ASTM Standard Specification and Test Method for Metallic Bone Plates (11), a support span of 90 mm was used, and bending/flexure testing was performed at a quasi-static loading rate of 5 mm/min until plastic deformation occurred (Figure 4). Plates were oriented with the tension surface facing downward. Plastic deformation was identified from the load–displacement curve. Although three-point bending inherently generates a bending moment at the loading anvil, care was taken to prevent additional unintended bending moments by ensuring plate seating flush against supports and maintaining symmetric alignment within the fixture.

The area moment of inertia (AMI), a key geometric parameter influencing resistance to bending, was estimated from experimental bending data using the relationship I = EI/E, where bending structural stiffness (EI) was derived from three-point bending results obtained in the study, and E was assumed for 316LVM stainless steel. These values represent effective back calculated AMIs derived from the overall mechanical response of each individual plate construct rather than direct geometric cross-sectional measurements. Although the plates tested were identical, minor variation in experimentally derived AMI values may occur secondary to differences in the measured flexural stiffness, and inherent variability of experimental testing conditions. These AMI values are presented for descriptive comparison only.

Screw push-out force, flexural stiffness, flexural modulus, flexural strength, and maximum load values were extracted from load–displacement curves. No statistical comparisons were performed between bending test results due to the same plate type being tested.

## 3 Results

### 3.1 Push-out force testing

Screw push-out testing was completed for 31 screws across three plates (Plate 1–3). Mean ± SD push-out forces were Plate 1 = 2,839 ± 471 N; Plate 2 = 3,119 ± 274 N; Plate 3 = 3,018 ± 274 N (Table 1). Exploratory one-way ANOVA performed using individual screw push-out measurements identified no significant difference among the three tested plates (*F*(2,28) = 1.666, *p* = 0.207).

**Table 1:** Screw push-out force results (*N*) for three 3.5-mm combination-hole locking plates (InVictos). Results are presented as mean ± standard deviation (SD) and number of screws (*n*).

Plate	Mean +/− SD (*N*)	Number of screws (*n*)
1	2839.09 ± 470.75	11
2	3119.06 ± 274.44	10
3	3018.44 ± 274.29	10

### 3.2 Three-point bending testing

Flexural responses demonstrated similar behavior among plates, with maximum flexural strength ranging from 641 to 697 MPa, flexural modulus from 55,655 to 63,732 MPa, flexural stiffness from 133 to 141 N/mm, and maximum load to failure from 0.63 to 0.65 kN (Table 2).

**Table 2:** Flexural properties of three, 12-hole 3.5-mm combination-hole locking plates (InVictos) determined by three-point bending.

Plate	Max flexural strength (MPa)	Flexural modulus (MPa)	Flexural stiffness (N/mm)	Max force (kN)
1	641.20	56478.66	141.41	0.64
2	697.04	63732.01	136.08	0.65
3	643.36	55654.94	133.12	0.63

The means and standard deviations for max flexural strength, flexural modulus, flexural stiffness, max force, max screw push-out force, and calculated AMI for Plates 1–3 tested here are included in Table 3, in addition to published data for nine different veterinary locking plates. No statistical analysis was performed due to differences in testing methodology; however, qualitative comparisons can be made.

**Table 3:** Mechanical properties of 3.5-mm combination-hole locking plates (Plates 1–3) tested here, with literature values of veterinary locking plate systems provided for contextual reference.

Plate/study	Max flexural strength (MPa)	Flexural modulus (MPa)	Flexural stiffness (N/mm)	Max force (kN)	Max screw push-out force (kN)	Estimated AMI (mm^4^)	Testing set-up
Plate 1	641.20	56,478.66	141.41	0.64	2.84 ± 0.47	38.0	3-point bending with 90 mm support span
Plate 2	697.04	63,732.01	136.08	0.65	3.12 ± 0.27	32.4	3-point bending with 90 mm support span
Plate 3	643.36	55,654.94	133.12	0.63	3.02 ± 0.27	36.4	3-point bending with 90 mm support span
LCP 3.5 mm (4)	–	–	60.9 ± 12.6	0.112	–	32.9	4-point bending bone model with 25 mm fracture gap
ALPS-10 (4)	–	–	39.6 ± 2.45	0.058	–	Not calculable from published data	4-point bending bone model with 25 mm fracture gap
ALPS-11 (4)	–	–	74.9 ± 3.32	0.131	–	Not calculable from published data	4-point bending bone model with 25 mm fracture gap
SOP (4)	–	–	76.0 ± 6.89	0.141	–	Not calculable from published data	4-point bending bone model with 25 mm fracture gap
Fixin (4)	–	–	46.0 ± 1.47	0.074	–	24.9	4-point bending bone model with 25 mm fracture gap
PAX 3.5 mm polyaxial (2)			104 ± 6.18	0.66 ± 0.031	1.53 ± 0.30	31.9	4-point bending bone model with 25 mm fracture gap
PLS-1 polyaxial	–	–	360.3 ± 113.4	1.62 ± 0.25	2.28 ± 0.30	35.4	Axial compression bone model with 34 mm fracture gap
PLS-2 fixed angle	–	–	377.3 ± 109.3	1.34 ± 0.22	4.94 ± 0.89	30.5	Axial compression bone model with 34 mm fracture gap
Stainless steel polyaxial 3.5 mm with plugs (Evolox) (6)	–	–	92.0 ± 6.5	0.699 ± 0.064	–	Not calculable from published data	4-point bending bone model with 25 mm fracture gap

Values include maximum flexural strength (MPa), flexural modulus (MPa), bending stiffness (N/mm), maximum force at failure (kN), screw push-out force (kN), and testing method. Estimated AMI values represent effective values derived from bending data and are not directly equivalent to geometric AMI. AMI values for comparator plate systems were obtained from previously published studies or estimated from available manufacturer data (4, 12, 23, 24). Data are presented as mean ± standard deviation. All units were converted when necessary to ensure consistency across studies and differences in testing methodology.

LCP, locking compression plate; ALPS, advanced locking plate system; SOP, string of pearls; PAX, polyaxial locking system; PLS, pearl-type locking plate system.

## 4 Discussion

This study provides the mechanical properties of a 3.5-mm polyaxial combination-hole locking plate (InVictos), focusing on screw push-out resistance and three-point bending behavior under controlled experimental conditions. The results represent descriptive mechanical properties rather than comparative superiority, as direct statistical comparison with previous studies cannot be made due to differences in testing methodology, plate geometry, and construct configuration.

The push-out forces observed (2.84–3.12 kN) fall within the range of previously published values for other 3.5-mm polyaxial and fixed-angle systems; however, comparisons must be interpreted cautiously because prior studies employed different screw angulations, torques, and fixture configurations. For example, some studies tested screws at multiple angulations, while others tested constructs incorporating synthetic bone (2, 12). These differences prevent direct equivalence between datasets and limit conclusions to qualitative observations only. In the present study, screws were inserted with the intention of achieving 0° angulation; however, insertion was performed manually without a guiding jig or computer-assisted technologies, and minor angular variability cannot be excluded.

Variability in push-out force was observed among screws and may reflect both inherent differences in the screw–plate interface and progressive deformation of the plate during sequential testing. Since multiple screws were tested within the same plate, localized plastic deformation of the plate and threaded regions may have influenced the mechanical response of subsequent tests. This may affect both thread engagement and the surrounding plate structure, contributing to variability in measured holding strength.

The bending data (flexural strength 641–697 MPa; stiffness 133–141 N/mm) are consistent with expectations for stainless-steel plates tested under three-point bending. Bending behavior is influenced by both material properties and geometric factors, including the area moment of inertia. The use of 316LVM stainless steel likely contributed to the observed stiffness and strength, as stainless steel implants have a higher modulus of elasticity compared to titanium alloys, resulting in greater construct rigidity (20).

Interpretation of bending results must consider the limitations of the testing configuration. Three-point bending concentrates stress beneath the loading anvil. The reported stiffness values will differ from those generated through four-point bending, which distributes stress over a larger region (21). The difference usually results in slightly higher bending stiffness and lower localized stress in four-point bending models when comparing to three-point bending models for the same plate, which needs to be considered when comparing our experimental plates for bending stiffness and max force to values across the other studies. A three-point bending model was chosen for this study due to the ductile and homogenous construct of the plate and combination holes.

Previously published data provide a general context for interpretation of the present findings (Table 3) (2, 4, 6, 12). In the current study, mean screw push-out forces ranged from approximately 2.84 to 3.12 kN. Bufkin et al. (2) reported lower mean screw push-out forces for the PAX polyaxial locking plate (1.53 ± 0.30 kN); however, direct comparison is limited by differences in screw insertion torque, angulation, and test configuration. Tremolada et al. (12) reported variable screw push-out forces for polyaxial and fixed-angle constructs (2.28 ± 0.30 kN and 4.94 ± 0.89 kN, respectively), further illustrating the influence of screw–plate interface design and testing methodology on measured outcomes. Similarly, Kaczmarek et al. (7) reported progressive reductions in screw holding strength with increasing off-axis insertion angles in both fixed angle and variable angle locking systems. Polyaxial systems demonstrated greater sensitivity to insertion angle, suggesting that variable-angle thread engagement may be more susceptible to cross-threading rather than a true locking interface, resulting in decreased screw push-out strength (22). When considered qualitatively, the push-out forces observed in the present study fall within the range of values reported for other veterinary locking plate systems, highlighting expected variability across implant designs and experimental conditions. Exploratory one-way ANOVA performed using individual screw push-out measurements identified no significant difference among the three tested plates (*F*(2,28) = 1.666, *p* = 0.207). Although a total of 31 screw push-out tests were performed, only three plates were evaluated. Therefore, the statistical analysis should be interpreted cautiously because multiple screw measurements originated from the same plate and are not fully independent observations. The ANOVA was included as an exploratory assessment of consistency among tested specimens. The absence of statistically significant differences among plates further supports the reproducibility of screw–plate interface performance under the testing conditions used in this study. Additionally, the screw push-out forces in the present study indicate that the combination-hole design did not compromise locking screw retention, which is the primary mechanical objective of evaluating the locking interface.

Interpretation of bending performance across studies requires careful consideration of differences in loading mode, stress distribution, and failure mechanisms. In the present study, three-point bending yielded flexural stiffness values of 133–141 N/mm and maximum loads to failure of 0.63–0.65 kN. Blake et al. (4) reported lower bending stiffness (39.6–76.0 N/mm) and maximum force (0.058–0.141 kN) for several standard locking plate systems tested under alternative experimental conditions. Bufkin et al. (2) reported bending stiffness of 104 ± 6.18 N/mm and maximum force of 0.66 ± 0.031 kN for the PAX locking plate, while Tremolada et al. (12) demonstrated higher stiffness and maximum force values for pearl-type polyaxial and fixed-angle constructs using different test configurations. Viitanen et al. (6) reported bending stiffness of 92.0 ± 6.5 N/mm and maximum force of 0.699 ± 0.064 kN for a stainless-steel polyaxial plate with locking plugs. The bending stiffness and maximum load values of the plates tested here suggest that the combination-hole configuration did not adversely affect bending behavior within the context of three-point bending. This data indicates that the bending properties of veterinary locking plates vary substantially across implant designs and testing methodologies. The results of the present study are therefore best interpreted as descriptive mechanical characteristics that fall within the spectrum of previously reported values rather than as evidence of superior mechanical performance.

The area moment of inertia (AMI) was estimated to provide additional geometric context for bending behavior. Back- calculated effective AMI values (32.4–38.0 mm^4^) fall within the range previously reported for 3.5-mm locking plate systems. Although all tested places were identical in nominal dimensions and configuration, these values represent experimentally derived effective AMIs derived from measured bending response rather than direct geometric measurements. Minor variations among plates reflect experimental variability, localized deformation at the combination-hole interface, and differences in measured flexural stiffness rather than true geometric differences between plates. Direct comparison remains limited because the present values were experimentally back-calculated from bending response, whereas previously published values were obtained using differing geometric or structural analysis available from the literature or manufacturer CAD data.

Although a statistical comparison of AMI across plate systems could be considered, this was not performed because AMI values were estimated rather than obtained from experimentally derived data. For this reason, descriptive comparison was considered most appropriate. Additionally, AMI values could not be determined for all plate systems included due to the lack of available geometric or structural data included in the literature or manufacturer CAD data. This incomplete data further limits cross-study comparisons and reinforces that AMI should be interpreted as another descriptive parameter rather than a variable suitable for direct statistical comparison.

This study has several limitations. Only three plates were tested, limiting generalizability. Push-out testing was performed at the intended 0° angulation to establish a standardized baseline and minimize effects associated with screw angulation. Additionally, screw insertion was performed manually, and minor angular variability cannot be excluded, particularly given the geometry of the combination-hole design. Therefore, the reported 0° angulation should be interpreted as the intended insertion angle rather than a mechanically constrained condition. Push-out testing was also performed using a torque higher than AO recommendations, which may influence holding strength. Three-point bending was selected for construct simplicity and alignment with ASTM testing standards; however, it characterizes baseline flexural performance under controlled conditions and does not replicate physiologic bending environments or four-point bending behavior. Comparisons to historical data remain qualitative due to differences in plate materials, geometries, working lengths, and loading configurations.

Future studies should also evaluate cyclic fatigue testing and fracture gap or bone models to better replicate clinical conditions. Also, testing screw push-out at different angles would add important information for usage in angulation. In addition, high-resolution imaging techniques such as micro-computed tomography or scanning electron microscopy (SEM) may provide further information on deformation patterns and failure mechanisms to determine the relevance of biomechanical data to clinical practice.

Overall, the 3.5-mm combination-hole polyaxial locking plate (InVictos) evaluated in this study demonstrated consistent screw-plate interface holding strength and reproducible bending behavior under controlled testing conditions. The push-out forces and flexural properties fall within ranges of previously reported literature. Interpretation is limited by differences in testing methodology and construct configuration. These results describe baseline mechanical characteristics of the tested plates, and should not be interpreted as evidence of superior performance.

## 5 Conclusion

The 3.5-mm combination-hole locking plates (InVictos) evaluated in this study demonstrated screw push-out and flexural properties that fall within the range of values previously reported for veterinary locking plate systems.

## Data Availability

The raw data supporting the conclusions of this article will be made available by the authors, without undue reservation.
